# Probing Trace Elements in Human Tissues with Synchrotron Radiation

**DOI:** 10.3390/cryst10010012

**Published:** 2019-12-27

**Authors:** Mihai R. Gherase, David E. B. Fleming

**Affiliations:** 1 Department of Physics, California State University, 5241 N. Maple Avenue, Fresno, CA 93740, USA; 2 Physics Department, Mount Allison University, 67 York Street, Sackville, NB E4L 1E6, Canada

**Keywords:** synchrotron, X-ray fluorescence, X-ray absorption spectroscopy, trace elements, biological tissues, medicine, diseases

## Abstract

For the past several decades, synchrotron radiation has been extensively used to measure the spatial distribution and chemical affinity of elements found in trace concentrations (<few μg/g) in animal and human tissues. Intense and highly focused (lateral size of several micrometers) X-ray beams combined with small steps of photon energy tuning (2–3 eV) of synchrotron radiation allowed X-ray fluorescence (XRF) and X-ray absorption spectroscopy (XAS) techniques to nondestructively and simultaneously detect trace elements as well as identify their chemical affinity and speciation in situ, respectively. Although limited by measurement time and radiation damage to the tissue, these techniques are commonly used to obtain two-dimensional and three-dimensional maps of several elements at synchrotron facilities around the world. The spatial distribution and chemistry of the trace elements obtained is then correlated to the targeted anatomical structures and to the biological functions (normal or pathological). For example, synchrotron-based in vitro studies of various human tissues showed significant differences between the normal and pathological distributions of metallic trace elements such as iron, zinc, copper, and lead in relation to human diseases ranging from Parkinson’s disease and cancer to osteoporosis and osteoarthritis. Current research effort is aimed at not only measuring the abnormal elemental distributions associated with various diseases, but also indicate or discover possible biological mechanisms that could explain such observations. While a number of studies confirmed and strengthened previous knowledge, others revealed or suggested new possible roles of trace elements or provided a more accurate spatial distribution in relation to the underlying histology. This area of research is at the intersection of several current fundamental and applied scientific inquiries such as metabolomics, medicine, biochemistry, toxicology, food science, health physics, and environmental and public health.

## 1. Introduction

A synchrotron is a radio frequency (RF)-type electron-accelerating machine developed initially as a tool for research in nuclear and particle physics. Although the first synchrotrons were designed to accelerate electrons, it was later realized that the electromagnetic radiation emitted by bending multi-MeV electrons in a magnetic field could have important scientific applications [1]. This radiation is referred to, herein, as synchrotron radiation (SR), and covers a large portion of the electromagnetic radiation spectrum, from infrared to hard X-rays. Waves and particles are the two fundamental physics concepts describing the production, propagation, and interactions with matter of electromagnetic radiation. While the concept of electromagnetic waves dates back to the nineteenth century, the concept of light particles or quanta, called photons, was first introduced by Albert Einstein in 1905 [2] to explain the photoelectric effect—one of the few known phenomena in the early 1900s that did not fit in the classical physics framework. Scientific applications of SR utilize both concepts of electromagnetic radiation: (i) the particle-like properties (i.e., photons) consistent with fundamental photon-matter interactions such as the photoelectric effect and Compton scattering and (ii) the wave-like properties which are the basis of X-ray diffraction, crystallography, and phase-contrast X-ray imaging. The emission of electromagnetic radiation by electrically charged particles in an accelerated motion was known before 1900, but the detailed properties of SR were only theoretically derived in the 1940s [3,4]—the same time period of the first experimental observations [1].

Modern synchrotron facilities are built around a fixed-energy synchrotron machine called a storage ring. The storage ring optimizes the energy required to accelerate the electrons and maintain them in a magnetically-guided loop for a time in the 5–100 h range [5]. In a typical storage ring, the electrons are accelerated to a few GeV of energy, which is about three orders of magnitude larger than the 0.511 MeV electron mass energy (i.e., ultrarelativistic regime). Using the special relativity equation relating the total energy and the mass energy of a particle, one can compute that a 3 GeV electron travels at 99.999997% of the speed of light in vacuum. SR is generated when the ultrarelativistic electrons pass through dedicated periodic magnetic structures called wigglers and undulators [6]. The properties that set apart SR from other sources of electromagnetic radiation are: fine tuning of the photon energy over a wide range, high brilliance, extreme collimation of the photon beam, polarization, and time structure. The brilliance is perhaps the most important quality of SR and is defined as the number of photons per unit time, per unit source area, per solid angle, and per photon energy bandwidth [5]. Brilliance values of today’s synchrotrons in the X-ray region exceed 10^15^ photons s^−1^ mm^−2^ mrad^−2^ (0.1% bandwidth)^−1^ [7]. By comparison, these values are about thirteen orders of magnitude higher than the brilliance of the most performant rotating anode X-ray tube sources [8]. The very large number of spatially confined photons emitted each second is an essential feature of SR beams for the following reasons. First, it facilitates experimental observations and data acquisitions of processes occurring with a very low probability in a short or reasonable amount of time. In very broad terms, these phenomena include photon scattering and absorption events, and photon-induced quantum transitions in atoms, molecules, and condensed matter systems. Second, using dedicated X-ray optics instrumentation, X-ray beams of lateral sizes of a few nanometers to a few micrometers range, can be achieved [9]. The microbeam performance allows various microscopy studies at unprecedented spatial resolution. Third, manipulations of the SR beams, such as fine energy tuning for X-ray absorption spectroscopy (XAS) studies, are not particularly efficient, and, therefore, require very intense beams.

There are more than 50 synchrotron facilities worldwide spanning the third and fourth generations, where tens of thousands of users perform experiments for scientific and industrial purposes with a research activity accounting for a few thousand peer-reviewed papers each year [7], [10]. For the past 70 years, synchrotron-based research has transformed the conventional thinking of what is experimentally possible in large realms of the natural and applied sciences: material science and engineering, condensed matter physics, chemistry and biology-related sciences, medicine, geology, environmental sciences, and public health. More recently, paleontological, archeological, anthropological, and cultural studies also utilized SR [11-13]. In a review paper published almost 15 years ago, Bilderback et al. [5] acknowledged the success of the third generation SR applications to biological sciences as summarized in their brief research highlights subsection titled “Structural Biology”. With the research output of such a large number of facilities and synchrotron users, a comprehensive review of any given area of scientific investigations, even if narrow, is daunting.

In this paper, we draw the reader’s attention to biological and medical synchrotron studies, and, within this area, the focus will be even narrower, by including only research involving the distribution and biochemistry of trace elements in human tissues. Synchrotron-based biological and medical research including conventional and phase-contrast X-ray imaging (planar and tomographic modalities) [14-18], protein crystallography and X-ray diffraction (XRD) on cells and tissues [19-21], and radiation therapy studies [22-24] were excluded. The importance of such investigations for the advancement of both diagnostic and therapeutic modalities employed in clinical practice, cannot be underestimated. Literature reviews on these topics have been published and some can be found in our sample list of references which were mostly selected from the more recent papers with no guiding principle in mind. More studies and reviews will likely appear in the future with the growing interest in the unique experimental opportunities that SR offers in these fields of research.

A schematic exposition of the physics behind the X-ray fluorescence (XRF) and X-ray absorption spectroscopy (XAS) methods, a list of other synchrotron-based X-ray methods, and the topic of radiation-induced tissue damage, were grouped in the next section. Section 3 contains the bulk of this review. In each of the five sub-sections, the analysis of the published literature was organized by anatomical structures or systems: Section 3.1. Cell Biology And Cancer; Section 3.2. Brain And Nervous System; Section 3.3. Bone, Teeth, And Internal Organs; and Section 3.4. Hair, Skin, And Nails.

## 2. Preliminary Concepts and Ideas

### 2.1. XRF and XAS

In short, XRF indicates the elemental composition, while XAS provides insights into the chemical speciation and chemical bonding or affinity of a certain element. Employing appropriate calibration and analytical methods, XRF measurements can also estimate the relative or absolute concentrations of the identified elements. Detailed descriptions of the experimental and computational methods related to XRF and XAS techniques are beyond the scope of this article. The quantum mechanical theories of the electron transitions induced by the photoelectric effect will also not be presented here. Such descriptions and treatments can be found in dedicated textbooks and in research and topical review papers. Only the basic physics principles are presented briefly in the following paragraphs.

**Figure 1.**
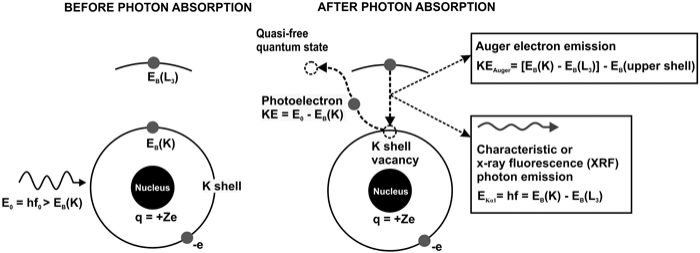
Before-and-after schematics of the photoelectric effect and associated transitions in an unspecified atom. For simplicity, only the initial photon, the characteristic or X-ray fluorescence (XRF) photon, the atomic nucleus, the two K-shell electrons, and one L_3_ sub-shell electron were drawn. The energy of each emitted particle was calculated as a function of the initial photon energy (E_0_) and the binding energies of the atomic electrons involved in the atomic transitions.

XRF and XAS rely on the physics of the photoelectric absorption process in which the energy of an incident X-ray photon is completely transferred to an atom. The probability of the photoelectric absorption event as a function of the incident photon energy and the byproducts of the atomic de-excitation transitions are the basis of the two analytical techniques. Figure 1 describes schematically the photoelectric effect and the associated phenomenology.

In Figure 1, the photon absorption is followed by the following atomic transitions: (1) the bound K-shell electron (called photoelectron) is ejected to a quantum state outside the atom; (2) the K-shell vacancy is filled by an electron from the L_3_ sub-shell; (3) the energy loss of the L_3_ sub-shell electron prompts two competitive processes: (a) Auger electron emission which refers to the ejection of an electron in an upper sub-shell and (b) characteristic or XRF photon emission. The XRF photon energy and the kinetic energies of the photoelectron and the Auger electron can be written as a function of the electron binding energies involved in these transitions by employing energy conservation. These relationships indicate how XRF photon and Auger electron emissions identify unique atomic transitions and, therefore, the atoms themselves. Several notes regarding the XRF and the Auger electron emissions are included in the next few paragraphs.

Linear momentum conservation also holds—initial photon momentum is transferred to the photoelectron and the photon-absorbing atom. However, the kinetic energy transferred to the atom is negligible due to the large mass difference between the electron and the atom. The vacancy in a shell or sub-shell prompts a host of electron transitions from the electrons in the upper sub-shells with varying probabilities, with the X-ray emissions prompted by a K-shell vacancy being the most intense. These electron transitions, linked to the XRF and Auger electron emissions, are identified by the old Siegbahn or the new International Union of Pure and Applied Chemistry (IUPAC) notation system. As an example, in the schematic of Figure 1, the K_α_1__ Siegbahn notation corresponds to the K-L_3_ notation in the IUPAC system. Despite the fact that IUPAC notation clearly identifies the atomic shells and sub-shells of the atomic transitions, the Siegbahn notations are still frequently used in many XRF studies.

The excitation energy corresponding to the initial atomic vacancy is not dissipated in a single electron transition such as the one depicted in Figure 1. The initial electron transition induces vacancies in the upper sub-shells and the chain of electron transitions followed by the combined XRF and Auger electron emissions continues until the atom reverts to its original ground state.

For lower atomic number elements, the Auger electron emissions dominate the XRF photon emissions, and the opposite occurs in heavier atoms. Measurements of the Auger electron kinetic energy spectrum (KE_Auger_) can be used to identify elements in a similar fashion to XRF and forms the basis of Auger electron spectroscopy. However, Auger electrons have kinetic energies in the range of a few eV to a few tens of keV which is easily dissipated in a large number of electromagnetic interactions with the electrons and nuclei of ordinary matter. Electron path in matter is short and tortuous (i.e., large scattering angles). For example, the range of a 20 keV electron in air is only 10 μm [25]. Analytical applications of Auger electron or photoelectron spectroscopy are, thus, limited to surface analysis, require high-vacuum conditions, and a monoenergetic X-ray photon irradiation. Hence, instrumentation dedicated to such spectroscopy measurements is typically found at synchrotron facilities and, in general, not compatible with in vitro or in vivo measurements of trace elements in tissues.

**Figure 2.**
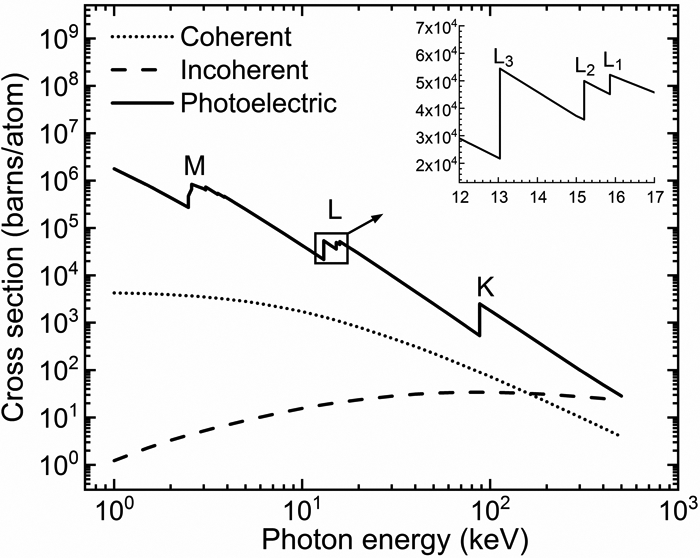
Plot of the X-ray photon coherent, incoherent (Compton), and photoelectric cross sections of Pb in the 1 keV to 500 keV energy range. The main log–log plot indicates the strong dependence of these interactions on the photon energies. The sub-plot shows the edges of the three L sub-shells. The upper-case letters in the main plot indicate the edges corresponding to the K-, L-, and M-shells. In the quantum mechanical theory of the atom, these shells are identified by the principal quantum number *n* values of 1, 2, and 3, respectively.

The photon energy for which photoelectric absorption occurs resulting in a vacancy in a particular shell or sub-shell, is called the K-edge for K-shell electrons, L_1_-edge for L_1_ sub-shell electrons, etc. The edge energy is slightly lower than the corresponding atomic electron binding energy, the small difference accounting for the fact that the photoelectron does not transition to an entirely free quantum state (i.e., null binding energy at an infinite distance from the atom). The photoelectron emission and initial vacancy can also occur in sub-shells of the upper L, M, or N atomic shells. The probability of a vacancy in the sub-shell of an atom can be computed from the photoelectric cross section dependence on the photon energy. The textbook by Podgoršak [26] is a useful reference on this topic and includes sample calculations of the K shell and L and M sub-shell vacancies in the lead (Pb) atom. The incident photon can also undergo coherent (or elastic) and incoherent (or Compton) scattering events. These interactions need to be accounted for in XRF and XAS measurements and subsequent data analysis. The cross sections (in units of barns per atom) for each of the three photon interactions as a function of the photon energy (E) for the Pb atom in the wide energy range from 1 keV to 500 keV, are drawn in the plot of Figure 2 below. The data was taken from the XCOM: photon cross sections database of the National Institute of Standards and Technology (NIST) [27].

One can observe that on the X-ray photon energy range up to and slightly above the K-shell electron binding energy, the photoelectric absorption dominates over the X-ray photon scattering interactions—an essential fact, valid for all atoms, and the physical basis of many X-ray applications including XRF and XAS. For photon energies much larger than the atomic electron binding energies, the ranking of interaction probabilities can be deduced from the trend above 100 keV photon energy in the Figure 1 plot. The probabilities of coherent and photoelectric interactions decrease, while that of Compton scattering events increases. For photon energies much higher than the 1.022 MeV value (twice the electron mass energy of 0.511 MeV), photon interactions are dominated by electron-positron pair production. In this context, it is perhaps relevant to note the energy scale difference between the ~50 eV or lower values above the shell or sub-shell edge of XAS spectroscopy measurements and the keV scale of characteristic photon energies in XRF spectroscopy. XAS—described qualitatively in the paragraph below—exploits the variations of the final quantum states of the photoelectron induced by the chemical bonds of the absorbing atom. XRF identifies chemical elements by exploiting intrinsic interatomic differences of core electron binding energies.

The physics behind XAS is briefly described in this paragraph by shifting the attention to the photoelectron in its transition from the bound atomic orbital to the quasi-free quantum state, as depicted in the schematic of Figure 1. If the incident X-ray photon energy is increased incrementally in small steps on the order of 1 eV from just below to above the edge energy of the atomic shell or sub-shell, the kinetic energy of the photoelectron will increase by the same amount. At lower kinetic energies, the photoelectron can transition to unoccupied higher atomic orbitals, and at higher kinetic energies, can scatter off the neighboring atoms. Hence, the final quantum state of the photoelectron depends on the electronic configuration surrounding the atom shaped by the types of neighboring atoms, inter-atomic distances, or the type of chemical bonds. The probability of such transitions obeys Fermi’s Golden Rule, first derived by Dirac [28] almost a century ago. Accordingly, the transition probability depends only on the initial quantum state (photon and atom in ground state) and the final quantum state (atom with a vacancy and photoelectron). Therefore, measurements of the photoelectric absorption rate at photon energies above the edge can provide information regarding the type of atoms surrounding the X-ray absorbing atom, inter-atomic distance, and the type of chemical bonds. Two regimes are typically distinguished within XAS: (i) X-ray absorption near-edge structure (XANES) and (ii) extended X-ray absorption fine-structure structure (EXAFS). The acronyms indicate the proximity to the edge energy of the spectral features, and, by employing the previous electron energy argument, the spatial extent of the photoelectron scattering events. The spectral features of XANES spectra are determined by the quantum mechanical amplitudes of photoelectron’s multiple backscattering events off the neighboring atoms, while EXAFS spectra features are mostly determined by the amplitudes of single scattering events.

Chemical bonding information and chemical affinities of elements are, in general, known. However, the same chemical element can exhibit multiple chemical affinities and propensity to bond to various molecules or molecular groups. These various chemical forms are called chemical species of the element and are often encountered in biological structures. Determination of the chemical species of an element in a sample and their relative fractions is called speciation. XAS techniques are physical methods employed to acquire XANES and/or EXAFS spectra. Suitable analysis of the XAS spectra is then able to determine speciation in complex chemical environments such those encountered in biological samples. Experimental XANES spectra of a targeted element are compared with standard XANES spectra obtained from compounds in which the element is present in known chemical structures. The procedure can provide the element’s speciation. Qualitative comparisons can just identify chemical species. Quantitative analysis yields measurements of the identified chemical species’ fractions. Fourier analysis of EXAFS spectra can determine the number of the neighboring atoms and their distances from the X-ray absorbing atom [29].

**Figure 3.**
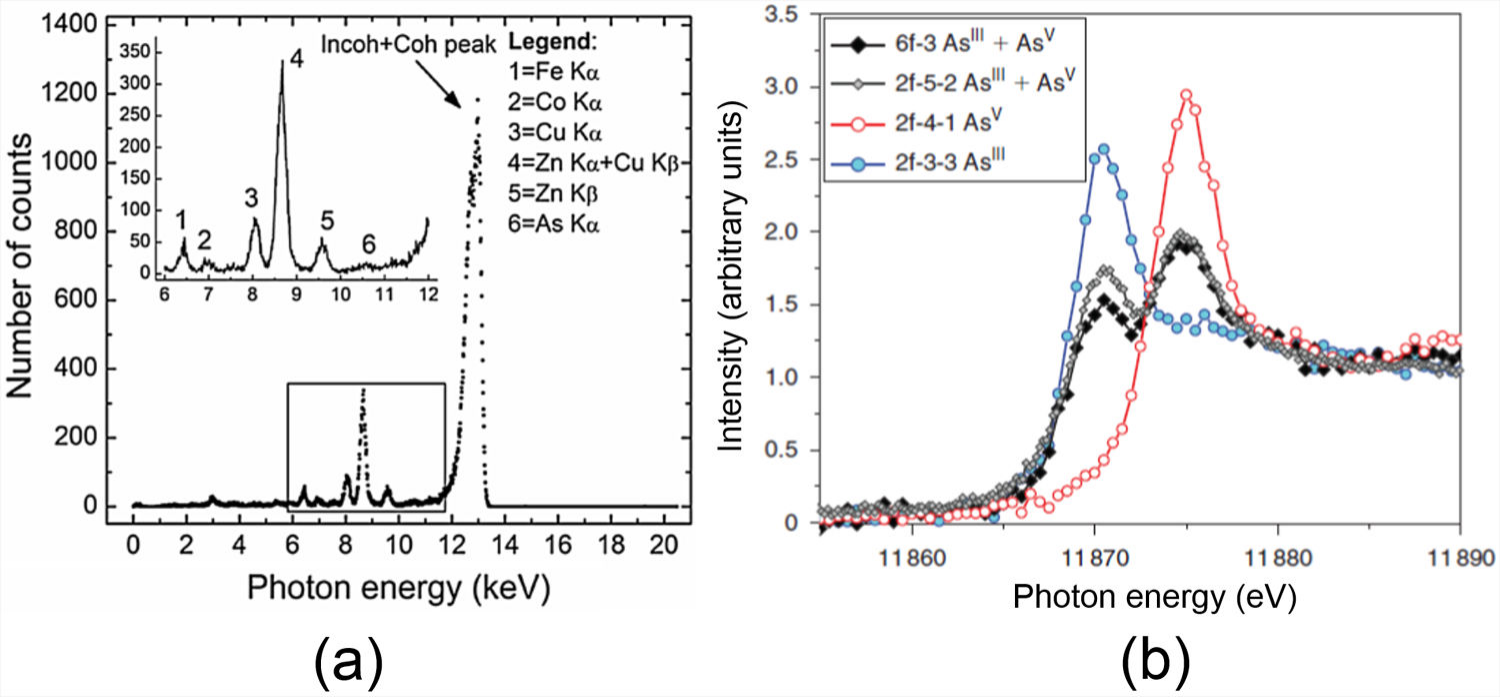
(**a**) XRF spectrum obtained from a 260-μm thick human fingernail cross section following a 2-s irradiation by a 13.0 keV monoenergetic X-ray beam at the Hard X-ray Micro-Analysis (HXMA) beamline from the Canadian Light Source (CLS) synchrotron. (**b**) X-ray absorption spectroscopy (XAS) spectra of As in four different human fingernail cross sections highlighting the pure and mixed As oxidation states. The red data points correspond to a high As concentration area in the same fingernail sample as the X-ray fluorescence (XRF) spectrum shown in plot (**a**). XAS spectra were acquired at the CLS and the Stanford Synchrotron Radiation Lightsource (SSRL) synchrotrons.

Examples of XRF and XAS spectra are shown in the plots of Figure 3a,b, respectively. The spectra were taken from two different synchrotron-based studies: Gherase et al. [30] and Ponomarenko et al. [31] and targeted As microscopic distribution in human nails and the corresponding As speciation, respectively. Both studies used cross sections of human fingernail clippings from volunteers living in the vicinity of Sackville, New Brunswick, Canada. The emitted XRF photons form the observed Gaussian peaks in the spectrum shown in Figure 1a. The XRF photon energies are on the keV scale and can identify the elements in the sample in concentrations as low as a few μg/g. By applying appropriate calibration and analysis, spectral data can also provide quantitative information expressed as relative elemental concentrations or absolute elemental mass concentrations.

In contrast, XAS spectra, indicated by the colored data points and connecting lines in the plot of Figure 3b, measure the relative photon absorption probability as a function of the incident photon energy. Using a double crystal monochromator geometry, the incident X-ray photon energy is varied in small steps on the eV scale around the As K-edge (~11.87 keV) as indicated by the *x*-axis values in the XAS spectra plot. The variation which can be visually observed in the four XAS spectra in the plot of Figure 3b is due to presence of one or two of the As oxidation states (III and V). This, in turn, can be linked to the binding of As to various chemical groups as will be discussed in Section 3.4.

In general terms, XRF and XAS provide complementary information. XRF can indicate, not only the presence of elements, but XRF maps measure spatial micro-distributions which indicate elemental accumulation. Although chemical affinity cannot be determined directly from XRF measurements, it can be inferred from data analysis of XRF maps for certain biomolecules or molecular families. Chemical affinity can be further linked to molecular mechanisms underlying biological processes at all scales. XAS brings specificity to the XRF data by determining the various oxidation states and chemical binding of a certain element. The human nail As XAS example in the previous paragraph highlights the complexity of chemical binding in biological systems, and even simple tasks, such as isolating the dominant chemical affinity, are not trivial.

In a general discussion of XAS and XRF applications to trace elements detection and chemical characterization for biological and medical research, it is also worth mentioning the competing method using fluorescent molecular probes. The method consists in the change of optical fluorescent properties of a molecule following binding to a certain metal as explained in the review by Terai and Nagano [32]. The immediate advantage of such approach is the use of well-established fast optical microscopy techniques to measure elemental distribution in tissue with a high signal-to-noise ratio (SNR) if attenuation of visible light in tissue is negligible. However, there are several important drawbacks such as: (i) simultaneous measurements of several trace elements is problematic due to high selectivity of fluorescent molecules; (ii) dependence of the fluorescent probe’s chemical affinity on the chemical form of the targeted metal as highlighted by Pushie et al. [33] for Hg, Cu, and Zn; (iii) tissue depth limitation imposed by the absorption of the emitted fluorescence light; and (iv) availability of biocompatible fluorescent molecular probes for all biologically-relevant trace elements.

Limitations of XRF and XAS techniques lie at the intersection of several physical, biochemical, and instrumentation factors. The performance of both XRF and XAS methods are negatively affected by X-ray photon scattering. For a given experimental setup and acquisition procedure, X-ray scattering is the main physical factor determining detection limits of trace elements in XRF and spectral quality in XAS. Significant reduction of backscattering events occurs in the limit of the total X-ray reflection regime. At incidence angles below the so-called critical angle *θ_c_*, X-rays are mostly reflected at an angle equal to the incidence angle, thus drastically reducing the probability of backscattered events and, consequently, improving elemental detection limits to concentrations in the 10^−12^ g/g to 10^−9^ g/g range [33]. However, *θ_c_* is inverse proportional to photon energy, hence, it is even smaller for increasingly higher X-ray photon energies. The geometrical constraints imposed by the very small critical angle have limited total reflection X-ray fluorescence (TXRF) or XANES applications to probing elemental composition of small samples or with very smooth surfaces [34]. Spatial resolution of XRF and XAS elemental mapping depends mostly on the X-ray beam size which has been decreased by continuous advancements in the X-ray optics field at all photon energies. Data acquisitions of larger tissue samples at high spatial resolutions are typically long, from tens of minutes to several hours or longer. This can be effectively reduced in the future by increasingly brighter SR beams matched by faster detectors characterized by high detection efficiency at all XRF photon energies: low attenuating detector windows for X-ray photons with energies below 5 keV and thick Si layers for X-ray photons above 20 keV. Long acquisition times are not only impractical, but the associated high X-ray exposure of tissues also poses risks of radiation damage which require awareness and mitigation strategies as briefly discussed in Section 2.3.

### 2.2. Additional Synchrotron-Based X-ray Methods

In addition to XRF and XAS, probing trace elements and their roles in biological tissues also uses other measurement methods and techniques using the important properties of the X-ray SR beams mentioned earlier: (i) monochromaticity, (ii) collimation, (iii) polarization, and (iv) coherence. These methods were used to support or augment XRF and XAS data in some of the scientific investigations included in this paper. A detailed technical presentation of these X-ray methods is beyond the scope of this review and recent advances in such methods and instrumentation can be found in dedicated reviews. Table 1 shown below is a list of these methods briefly describing their corresponding underlying physical principles and recent published reviews on these topics. Measurement methods not involving SR X-ray beams were not included in Table 1, but, where applicable, were specified together with their scientific contributions in each reviewed study.

### 2.3. Radiation-Induced Damage in Biological Samples

With ever brighter and more collimated synchrotron X-ray beams, the issue of radiation-induced damage in tissues requires careful planning and design of the XRF or XAS experiments involving biological samples. Ongoing research is centered on reducing the X-ray exposure and establishing efficient ways to reduce the radiation-induced damage without affecting the accuracy and outcome of the intended measurements. First, it is important to note that the absorbed doses in SR-based studies of biological samples are extreme. Although radiation-induced damage can occur at doses of just a few grays (1 Gy = 1 J of absorbed radiation energy per 1 kg of absorbing tissue mass), the X-ray exposures in synchrotron-based studies reach the MGy (10^6^ Gy) unit scale and higher [41,42]. By comparison, radiation doses from exposures to X-rays or gamma-rays in clinical procedures are below 1 Gy and are measured in units of cGy (10^−2^ Gy) in external-beam radiation therapy, and in units of mGy (10^−3^ Gy) in conventional diagnostic X-ray imaging and nuclear medicine procedures [43]. Second, radiation-induced damages cannot always be detected by quick XAS scans, and can significantly alter experimental outcomes.

Atomic ionizations and excitations induced by the primary X-ray photon interactions and secondary electrons can trigger chemical reactions which are the main source of the radiation-induced damage. These studies may, therefore, be included in the wider area of photochemistry which considers chemical effects of light. However, there are important differences amongst the interactions leading to chemical effects across the electromagnetic spectrum. Research of radiation-induced chemistry and their side effects spans several decades and a review is beyond the limited purpose of this sub-section. Radiobiology, the study of the interactions of radiation with living systems and the basis of modern radiation treatments of cancer, along with space science and technology, are perhaps the most known applications of this branch of chemistry. A detailed account of photo-reduction reactions in biological XAS studies can be found in the review by George et al. [44], while Garman and Weik [42] highlighted some recent research work on assessing radiation damage and mechanisms in DNA, amino acids, and proteins.

Water is a major component of many biological structures and it is often the solvent of biological molecules. Hence, its photochemistry is important in this context. The water radicals and ions formed during water ionization, and the cascade of reactions that follow, are responsible for indirect damage to other biomolecules, including nucleic acids. On a side note, water radiolysis is also important in other areas such as radiobiology [45], or calorimetric radiation dose measurements in water in which radiation-induced chemical reactions convert a fraction of the absorbed energy away from thermal energy [46].

Researchers have developed and employed various mitigation mechanisms which are summarized in reference [44] and references therein: (i) cryogenic methods (decreasing temperature reduces the rate of damaging chemical reactions), (ii) freeze-drying or lyophilization (decreasing the water content), (iii) free-radical scavengers (chemicals damping the effect of water free radicals such as glycerol, ethylene glycol, or sucrose), and (iv) electrochemical methods (counteracting photo-reduction reactions).

## 3. XRF and XAS Measurements in Human Tissues

### 3.1. Cell Biology and Cancer

With many advances in X-ray optics over the last three decades, synchrotron X-ray beams routinely achieve lateral sizes of a few micrometers and, over the past 15 years or so, nanoscale sizes are also becoming common at synchrotrons around the world [47-49]. This progress allowed scientists to foray into the role of metals and metalloids in biological processes taking place at the intracellular level. Such scientific explorations were difficult (specific dyes were used for microscopic localization of elements) or even impossible in the past [50]. It is estimated that about one third of all proteins contain metals, many of which are essential metalloenzymes. In recognition of the emergent research interest, Haraguchi [51] proposed in 2004 the term metallomics, to encompass all research fields focused on biologically-relevant metals.

Two directions of synchrotron-based SR-XRF and SR-XAS intracellular mapping emerged: (i) study of intracellular distribution of trace elements by association with known structures and (ii) probing cellular processes such as DNA transcription, translation, or apoptosis (i.e., cell death) by monitoring subcellular components labeled with metallic nanoparticles or engineered overexpression of metal-hosting proteins [50]. An example in the first category is the study of Munro et al. [52] aimed at probing intracellular mechanisms of arsenic (As) toxicity which is exploited in the antileukemia drug Trisenox (As_2_O_3_). In the study, HepG2 human hepatoma cells were treated with two As-based solutions abbreviated as arsenite and arsenate, which contained the trivalent As or As(III) and the pentavalent As or As(V), respectively. Using SR-XRF mapping at 0.3 μm spatial resolution, the authors determined that As accumulated in the nucleus of the HepG2 cells, more specifically in the euchromatin region of the nucleus in arsenite-treated cells. The euchromatin region comprises the most active DNA portion of the genome. This observation confirmed the suggestion that As cellular damage is caused by direct interactions with the DNA or with the DNA binding proteins. The maps also indicated localization of P, S, Cl, K, Ca, Zn, and Cu in both the control and the As-treated cells. Studying correlations amongst these elements and relating it to past studies, the authors determined or suggested other mechanisms related to As cell toxicity. For example, the high levels of Ca in the nucleus of As-treated cells indicated cell toxicity, and were possibly associated with mitochondrial-induced apoptosis, although the spatial resolution of the As maps could not indicate As damage or interaction to this cell structure. The analysis of XANES spectra demonstrated that the cellular As was bound to three S ligands corresponding to the As binding to the thiol groups of proteins. This conclusion was also supported by single-scattering analysis of the EXAFS recorded from cells treated with arsenite. Overall, the XAS part of the Munro et al. study [52] indicated that As toxicity originates in its propensity to bind to the DNA transcription proteins and not directly to DNA.

Subcellular XRF elemental mapping was also reported in the recent study by Yang et al. [53]. Using the European Synchrotron Radiation Facility (ESRF) nanoprobe capabilities, the authors employed XRF tomography combined with phase-contrast tomography to determine three-dimensional mass fraction maps of multiple elements at the subcellular level in normal and malaria-infested human red blood cells. Although phase-contrast X-ray techniques are not reviewed in this paper, it is important to note the use of multiple X-ray methods in recent studies. The trend will likely continue in the future, as multiple methods give complementary pieces of information which, when put together, can enhance the overall scientific value or applicability of the measurements. Two-dimensional XRF maps at 22 different angles between the sample and the incident beam were acquired covering 156°. Using these data, Yang et al. [53] were able to reconstruct quantitative three-dimensional elemental distributions of three elements: P, S, and Fe at the few hundreds nm spatial resolution level. The signal-to-noise ratio (SNR) and the root-mean-square error (RMSE) metrics were computed and presented for the five different tomographic reconstruction methods the authors employed. Out of these, the total variation minimization (TVM) tomographic reconstruction technique was found to give the highest SNR and the lowest RMES values. In addition to the tomographic techniques detailed in the paper, the authors highlighted the method’s performance by comparing the results extracted from the XRF data with published data from other microscopy techniques. The two-dimensional maps and slices of the three-dimensional XRF data which were presented clearly identified the malaria-infected cells by evidence of the Fe-rich area identified as the parasitic vacuole. This subcellular spatial concentration of Fe occurs through the digestion of hemoglobin and the crystallization of the hemozoin by the parasite which originates in the heme—the non-protein component of hemoglobin. Several quantitative results were found in good agreement with literature values such as the mass of the human red blood cell (~27.4 picograms) or the total number of Fe atoms in a human red blood cell (~ 9.0 × 10^8^). The authors also highlighted the superiority of the tomographic versus the conventional two-dimensional XRF approach. First, the data analysis was separated in the three distinct areas of the malaria-infested cells which were clearly identifiable in the elemental XRF maps: cell, parasite, and hemozoin crystal. The linear regression fittings of the tomographic data in the three intracellular areas yielded higher correlation coefficients between the S and Fe concentration measurements than the two-dimensional analysis. The results were consistent with existing knowledge on the molecular mechanisms of this parasite.

Another example of a XRF study with even better subcellular resolution is the recent study of Victor et al. [54] on *E. coli* bacteria cells. The authors acquired two-dimensional XRF, tomographic XRF, and ptychography measurements. Two-dimensional XRF maps indicated distributions of Cl, Ca, and Zn elements as well as the location of added 100 nm diameter Au nanoballs used as fiducial markers. Phase-contrast ptychographic images indicated the boundaries of both the individual cell and the nanoballs and the Zn tomographic data produced two-dimensional reconstructions that showed an uneven distribution of this important trace element throughout the *E. coli* cell.

Similar XRF studies that achieved elemental mapping at sub-cellular level were also reported by Vine et al. [55], Davies et al. [56], and Yan et al. [57]. One common denominator of these studies and the two discussed above, is the use of additional microscopy techniques and methods to complement or aid the XRF data. Vine et al. [55] used the simultaneous ptychographic modality to highlight the cell boundary (i.e., membrane), Davies et al. [56] compared their XRF results to laser ablation-inductively coupled plasma-mass spectrometry (LA-ICPMS) imaging, and Yan et al. [57] used coherent scattering data to reconstruct absorption- and phase-contrast images of the human chromosome. Another common denominator is the high radiation dose and scanning times associated with these techniques. Yang et al. [53] reported a radiation dose estimate of 7 × 10^8^ Gy and Victor et al. [54] reported 1.3 × 10^9^ Gy and a 15-hour time for the tomographic data acquisition of a single *E. coli* cell. Yan et al. [57] initially evaluated their study dose at around 10^8^ Gy, but subsequently estimated a much lower dose of about 4 × 10^5^ Gy when accounting for the large X-ray absorption of the silver (Ag) present in their samples which originated in the chromosome staining procedures.

The links between trace metals and cancer are manifold. First, several metals such as, Cd, Cr, and Ni are well-known and studied carcinogens [58]. All metals are now thought to promote cancer by a number of common mechanisms including redox reactions, methylation, altering gene regulation, and synergistic effects in the presence of other cancer-causing agents [59,60]. Second, abnormal concentrations of biologically important metals in cancerous tissues were observed in several synchrotron-based studies over the past decade. The biologically important trace element Zn is known to be a cofactor for more than 300 enzymes and an essential intracellular ion. SR-XRF was employed to look at Zn concentration in prostate cancer tissues (adenocarcinoma) [61] and breast tissue affected by ductal carcinoma [62]. The comparison with Zn concentration measurements in healthy tissues indicated a lower Zn uptake in prostate cancer tissues and a significantly higher Zn uptake in the ductal carcinoma tissues which was highly correlated to the elevated expression of estrogen receptor molecules.

A comparative approach between trace elemental concentrations in healthy tissues and cancer lesions was also employed in similar synchrotron-based studies targeting other biologically important elements including K, Ca, Fe, and Cu [63-68]. Elevated concentrations of these elements were observed in the cancerous lesions, however, the molecular mechanisms behind such observations were not always provided or clearly identified. XAS in combination or separate from XRF studies were employed to provide supporting data on the role of trace elements in the molecular mechanisms involved in cancer. The synchrotron-based XANES studies of Kwiatek et al. [69] and Al-Ebraheem et al. [70] examined the oxidation state of Fe in prostate cancer and invasive ductal carcinoma breast tissue samples, respectively. Their results appear to indicate that the fraction of Fe (III) oxidation state was slightly larger than the Fe(II) oxidation state. Both studies linked their findings to the known Fenton reactions between Fe(II), Fe(III), and reactive oxygen species. The reactive oxygen species can induce DNA damage and ultimately carcinogenesis. Al-Ebraheem et al. [61] mentioned, however, that the shift from Fe(II) to Fe(III) also occurs in normal tissues, therefore, it cannot be determined if the shift is the cause or the result of the carcinogenesis.

The XAS studies by Al-Ebraheem et al. [70], Podgórczyk et al. [71], and Weekly et al. [72] also probed the of oxidative states and chemical species of Zn, Zn and Cu, and Se, respectively, in tissues and cells affected by cancer. Both Zn and Cu XANES spectra in cancerous tissues indicated possible abnormal molecular mechanisms which might involve these elements, but the data did not support any conclusions in the absence of quantitative results. The metabolism of selenite $\left({\text{SeO}}_{3}^{2-}\right)$ in human lung cancer cells was probed over a 72 h period using EXAFS and XANES [72]. The results of this study indicated that the selenite metabolites likely increased the oxidative stress in the cells. The XRF maps showed localized Se in the cells’ cytoplasm and a two-fold increase of Cu accumulation. The XANES spectra indicated that the localized Se was mostly in elemental form while EXAFS spectra were consistent with Se─Se and Se─S bonding, but not with Se─Cu bonding.

An interesting study by Pascolo et al. [73] looked at the interactions of asbestos and Fe in lung tissue. The authors utilized soft- and hard-X-ray XRF and XANES at the ESRF to reveal some of the unknown carcinogenic and toxic mechanisms triggered in the lung tissues following human exposure to asbestos. The physical and chemical properties of asbestos and the Fe presence in its fibers make this material particularly toxic and a human carcinogen classified as such by the International Agency for Research on Cancer (IARC). The Fe content and the ability of very small fragments to attract Fe from the surrounding environment and form the so-called asbestos bodies are considered as the main mechanism of asbestos’ toxic and carcinogenic effects. The semi-quantitative analysis of the XRF microscopic images demonstrated that Fe levels in asbestos bodies were as much as 400 times higher than in asbestos-free blood vessels. The images also showed, for the first time, that the formation of the long-lasting asbestos body involves Fe-rich micro- and nano-aggregates in its proximity. Moreover, Fe mobilization and accumulation in the tissue surrounding the asbestos bodies is also accompanied by Mg aggregation and irreversible calcifications. The quantitative XANES analysis of Fe in asbestos bodies confirmed ferritin (blood cell protein) as the dominant Fe chemical form, however the concurrent presence in variable fractions of hematite (Fe_2_O_3_) suggested the alteration of Fe chemistry due to asbestos presence.

### 3.2. Brain and Nervous System

Subcellular and larger scale synchrotron-based XRF and XAS of the human brain and nervous system targeted neurodegenerative conditions such as Parkinson’s and Alzheimer’s diseases (denoted by PD and AD in this subsection). Using a rapid-scanning techniques (two speeds of ~22.8 ms and 5.7 ms per 40 μm-size step) Popescu et al. [74] acquired Fe, Zn, and Cu maps of normal and PD brain slices. The maps confirmed the known abnormal higher Fe concentration (by about 40%) in the *substantia nigra* pars compacta of PD brain compared to the normal brain. It is thought that the high Fe levels induce oxidative generation of free radicals which leads to the degeneration of dopaminergic neurons causing PD. The analysis of the obtained metal maps in PD brain slices also indicated low-concentrations of Cu areas co-localized with the very high Fe level areas and the XRF maps of the midbrain showed abnormal distributions of all three metals (Fe, Zn, and Cu) in the PD brain samples.

AD is a neurodegenerative disorder caused by the accumulation in the grey matter areas of the brain of senile plaques which are extracellular deposits of the amyloid beta (Aβ) protein and neurofibrillary tangles which are aggregates of the tau protein. The plaque formation is caused by the transformation of the Aβ protein from a soluble form (α-helix protein folding) to a neurotoxic fibrillary structure (β-sheet folding). Using SR-XRF and synchrotron-based Fourier transform infrared micro-spectroscopy (FTIRM), Miller et al. [75] demonstrated co-localization of higher Cu and Zn concentrations (using SR-XRF) with the regions of elevated β-sheet content (using FTIRM) in AD brain tissues.

Human brain tissues affected by AD were also studied from the perspective of Fe biochemistry. In particular, redox active Fe compounds such as magnetite (Fe_3_O_4_) were found in tissues from AD brain [76] and thought to have an important role in the progression of the disease. The distribution of Fe in AD brain was investigated in a synchrotron-based X-ray tomography study [77].

More recently, Fe co-localization with the Aβ protein and the formation of an Fe-amyloid complex was also demonstrated in an established mouse model of AD [78]. Soft X-ray XAS mapping using a scanning transmission X-ray microscopy (STXM) combined with the X-ray magnetic circular dichroism (XMCD) technique enabled the determination of the oxidation state as well as the magnetism of the Fe-based structures at the nanoscale level. The results of this study demonstrated that Fe deposits with different morphology and oxidation and magnetic states are not just localized to the regions of plaque-like deposition, but appeared to be an integral part of these structures. The authors also drew attention to the finding that diffuse amyloid deposits, which are typical of early-stage amyloid pathology, comprise a bound amyloid-Fe composite. This finding supports a model involving Fe in the genesis of the amyloid deposition in AD.

The complexity of amyloid plaques in AD which contain the Aβ protein, other proteins, lipids, and metal ions (Fe, Cu, or Zn) was also investigated by Summers et al. [79] by employing three different spectroscopic microscopy modalities: FTIRM, Raman microscopy, and XRF microscopy. By developing an experimental platform that optimized the inclusion of all three methods, the authors were able to study in a reasonable time frame the biochemical composition of the amyloid plaques by determining the aggregated protein content (FTIRM), total lipid esters (FTIRM), total lipid methylene groups (FTIR and Raman), total cholesterol (Raman) and Fe, Cu, and Zn (XRF) levels at sub-micrometer scale. Their results appear to indicate that elevated levels of aggregated protein, Fe, and Zn were found within the core of the amyloid plaque, while elevated levels of Cu and lipids, not including cholesterol, were found at the plaque periphery.

A slightly different subject of study on human brain pathology using SR-XRF is the paper by Robinson et al. [80]. The paper looked into the neurotoxic effect of manganese (Mn) exposure leading to manganism or Mn induced parkinsonism—a condition first diagnosed about 170 years ago. The authors used brain tissue samples extracted from Mn-exposed rodents exhibiting the neurotoxic symptoms of manganism to obtain elemental XRF maps of different regions of the brain. The quantitative analysis demonstrated that Mn distribution does not co-localize with those of Fe, Cu, or Zn. A high Mn concentration was also observed in the *substantia nigra pars compacta* structure of the brain—an area containing dopaminergic neurons in which neurodegeneration leads to PD.

### 3.3. Bone, Teeth, and Internal Organs

Synchrotron studies that focused on bone, teeth, and internal organs followed a similar pattern to the studies focused on other tissues. The observed microscopic elemental distributions were linked to pathologies or accumulations due to exposures. Having the largest mineral contents in the human body, bones, and teeth were the subjects of many synchrotron-based XRF studies over the past 10–15 years. Zoeger et al. [81] showed that Pb, following Ca metabolism, accumulates predominantly at the outer border of the cortical bone in human bone slices. The authors also measured a wide range (roughly from 0.03 to 0.4) of ratios between the Pb L-shell XRF signals measured in the cortical bone versus those measured in the trabecular bones at various anatomical locations. Simultaneous detection in the bone of other bulk and trace elements such as Ca, Zn, and Sr indicated a remarkable co-localization of Pb with Zn in cortical bone, but no mention was made on the origin of this observation.

More detailed synchrotron-based XRF data and more in-depth relationships with bone and joint anatomy, physiology, and histopathology were revealed and discussed in several papers published by an extended European research collaboration over the past 15 years [82-86]. A common denominator of these bone studies is the comparison of the synchrotron-based XRF elemental imaging and analysis with the images obtained from quantitative backscattered electron imaging (qBEI)—a scanning electron microscope (SEM) technique developed in the 1990s [87] which measured Ca concentration at a few micrometers spatial resolution based on a calibration with grey scale values from SEM images.

In the microscopic XRF studies of Zoeger et al. [82,83], the authors focused on the distribution of Ca, Zn, Sr, and Pb in human joint tissue thin slices. The main finding of these studies was that Pb and Zn accumulate within a few micrometers wide band at the tidemark—a histological structure at the boundary separating the non-calcified and the calcified regions of the cartilage known to play an important role in osteoarthritis. Zn is an essential element known to be involved in the normal growth of the skeleton and to accumulate in the tidemark and at sites of new bone formation. Regarding Pb the authors only speculated that Pb is known to interfere with Ca^2+^ signaling in cells, and, therefore, it might interfere with cation exchange processes in the hydroxyapatite crystal which could further affect the properties of this important component of bone mineral. The authors also indicated that the Pb distribution XRF measurements strengthen the conclusion of previous studies that Pb plays a role in osteoarthritis, with Pb toxicity targeting the osteoblasts (cells involved in bone formation) and chondrocytes (cells involved in the formation of the cartilaginous matrix).

The spatial distribution of the same four elements: Ca, Zn, Sr, and Pb was also investigated using synchrotron-based confocal micro-XRF (17 keV photon energy, 17 × 12 μm spatial resolution, and 19 μm depth resolution) and qBEI techniques in the study of Pemmer et al. [84]. The authors measured the relative XRF signal from these four trace elements in 14 human femoral bone samples collected from healthy and osteoporotic individuals. The important result of this study was that Zn and Pb had significantly higher concentration levels in the cement lines in comparison to the surrounding mineralized bone matrix. The relative measured differences also had a significantly wider variation for Pb than for Zn and Sr. The cement lines are histological boundary regions of increased mineral content and 10 μm thickness (2D sections of 3D cement surfaces) separating the old and the new bone matrix regions during bone remodeling process. This observation is consistent to the main finding in the previous study of Zoeger et al. [83], keeping in mind, however, the difference in anatomical locations between these two studies. The discussion section of Pemmer et al. [84] contained a detailed explanation of the observed differential accumulation amongst the three trace elements: Zn, Sr, and Pb. The elements can accumulate in the cement surface during the period this histological region is exposed to the interstitial fluid, until the new bone matrix is deposited by the osteoblasts. The reduced inter-sample variation of the Zn levels in the cement surface indicated that Zn is an intrinsic part of the bone remodeling process, being a metallic component of many of the enzymes regulating bone physiology, while the wider inter-sample distribution of Pb could be explained by fluctuations in the duration the cement surfaces were exposed to the interstitial fluid as well as intrinsic Pb concentration variations. Not included in this review, but supporting the experimental observations, Pemmer et al. [84] also discussed at length the biochemical mechanisms which involved the divalent positive ions of the four elements. No significant differences were observed between the healthy and osteoporotic bone samples, however, the authors pointed to the small sample number as an argument against drawing relevant conclusions.

Recent SR XRF studies also targeted osteosarcoma—the most common bone cancer in children and teens in which tumor cells produce immature bone also known as osteoid (the un-mineralized organic part of the bone matrix during bone formation). As reported by Rauwolf et al. [85] and Streli et al. [86], the Zn levels in the bone regions affected by osteosarcoma were found to be significantly higher than those in the healthy regions within the same bone sample. On average, the Zn fraction median in osteosarcoma regions, defined as the median of the relative counts ratio Zn/(Zn + Ca) distribution, was found to be about six times higher than that measured in healthy bone regions. Since Zn is known to be a stimulant of bone mineralization, the observed high Zn concentration level in poorly mineralized tumorous tissue seems counterintuitive. In the larger cancer research context, Rauwolf et al. [85] listed studies which reported decreased Zn levels in several types of cancer (a notable exception was the breast cancer study of Al-Ebraheem et al. [67]). As possible speculative explanations for the elevated Zn levels in osteosarcoma tissues, the authors pointed to: i) the possible effects of chemotherapy on Zn levels and ii) the reported low levels of Zn in serum bone turnover markers such as bone alkaline phosphatase in children and adolescents with osteosarcoma—an observation that would support the Zn accumulation in the bone tissue malignancies by depletion of Zn in the serum.

An interesting synchrotron-based XRF and XANES study by Ektessabi et al. [88] examined the distribution and chemical states of metals around total hip replacement implants. The prosthesis was made of a stainless steel head and titanium (Ti) alloy stem and cup which enclosed the head of the implant. Between the head and the cup alloys a polyethylene layer prevented direct friction between the two metallic alloys. In the patient under study, the wear of the polyethylene layer resulted in direct friction between the two metallic alloys. It was hypothesized that the friction led to the release of stainless steel and Ti nanoparticles. As a result, in the few tens of micrometers layer of tissue surrounding the head of the implant elevated levels of Fe, Cr, and Ni (elemental components of the stainless steel alloy) were determined by XRF raster scanning techniques (10 × 10 μm and 2 × 2 μm, both at 1 s per point measurement time). The geometrical pattern of the relative XRF intensity map of Fe suggested dissolution of this element from the diffused nanometer-size stainless steel particles—a conclusion also supported by the Fe XANES spectrum. The XANES spectrum of Cr supported the oxidative state (Cr_2_O_3_) rather than its pure metallic state, also consistent with a nanoparticle hypothesis which would promote Cr oxidation via a large surface area.

Trace elemental composition as a function of depth in the human tooth enamel was investigated in the studies by Guerra et al. [89,90] and by Wang et al. [91]. The main goal of these studies was the study of the tooth enamel as a biomarker for past Pb exposure, although the distribution of other trace elements was simultaneously assessed using SR XRF. In their initial study, Guerra et al. [89] used five incisor teeth from children in a neighborhood known for elevated Pb exposure with one 100 μm-thick slice from each tooth being analyzed. XRF raster scans of the tooth slices were obtained using a white beam with a lateral size of 20 μm and 12 s measurement time per point. The data analysis along the depth of the tooth indicated a large concentration (measured as relative XRF signal) of Pb in the superficial tooth enamel and significantly lower concentrations in the layers underneath the enamel: the dentinoenamel junction and the dentin. A later follow-up study by Guerra et al. [90] studied slices from five shed primary teeth of children from two different towns in different regions of Brazil, one of which was known for high Pb exposure levels from a lead ore smelter which operated between 1960 and 1993. Two teeth were screened as containing low Pb levels and labeled as “controls” while the other three were identified as originating from Pb-exposed children. Using a synchrotron beam of 20 μm lateral size and five points of 300 s measurement time, Guerra et al. [90] probed the distribution of Ca, K, Zn, Pb, Mn, Cu, and Sr in four layers of the tooth: superficial enamel (0 to 10 μm), subsuperficial enamel (10 to 30 μm), primary dentin, and secondary dentin. The relative XRF signals of the Pb relative to that of Ca indicated much larger concentrations of Pb in the superficial enamel layer, consistent with the group’s Pb concentration measurements of over 2 mg/g in the teeth of the Pb-exposed children using the inductively-coupled plasma mass spectrometry (ICP-MS) method. The shape of the relative elemental distributions also pointed to possible co-localizations of Zn, Pb, and Mn in the superficial enamel and Zn and Sr in the secondary dentin. The SR XRF study of Wang et al. [91] also looked at the Pb distribution in teeth and found slightly different results: in an incisor tooth sample the Pb distributed primarily in the secondary dentine region and only secondarily in the superficial enamel, while in the two molar tooth samples Pb was found primarily in the pulp and secondarily in the enamel. The authors also noticed two different co-localizations of the Pb: with Zn in the incisor sample and with Ca in the molar samples.

Excluding cancer-related studies, the authors did not come across many synchrotron-based studies centered on the distribution and the chemical state of trace elements in human internal organs. The number of such studies and their scientific inquiry areas will likely increase and broaden in the near future. We present the results of two studies in this area: nanoscale XRF imaging of environmental dust in human lung by Gianoncelli et al. [92] and the high Zn content of Randall’s plaque by Carpentier et al. [93].

Gianoncelli et al. [92] used human lung tissues collected post-mortem from three patients that lived in urban centers in the north of Italy with no known specific professional exposure. Using standard tissue staining and light microscopy methods, anthracosis (accumulation of carbon in lungs due to exposure to air pollution or inhalation of smoke or coal dust particles) regions were identified and 5-μm thick lung tissue sections were used for XRF mapping. Two XRF procedures were used: (i) 17.54 keV photon energy, a beam with a spot size of 60 × 60 nm, and 100 ms per point and (ii) 1.95 keV with a beam lateral size of 250 nm and a dwell time of 9 s per point. The low energy (1.95 keV) photons were used for optimal excitation of low-Z atoms such as Si, Al, and Mg and in scanning transmission X-ray microscopy (STXM) mode to generate absorption and differential phase contrast images at the sub-micrometer length scale. Using the 17.54 keV photon energy XRF procedure, maps of 16 elements (Si, P, S, K, Ca, Ti, Cr, Mn, Fe, Ni, Cu, Zn, As, Se, Br, and Sr) were obtained in three different microscopic regions varying in size: 34 × 34 μm, 12 × 12 μm, and 6 × 6 μm. Similarly, XRF maps of six elements (O, Na, Mg, Si, Al, and Fe) were obtained using the 1.96 keV photon energy as well as absorption and differential contrast phase images corresponding to regions of 20 × 20 μm and 16 × 16 μm sizes. The low-energy photon XRF elemental maps correlated well with both the absorption and differential contrast phase images: high-elemental concentrations regions corresponded to contrast features in the other two X-ray contrast mechanisms. The high-energy photon XRF maps identified nanoparticles of various sizes and elemental composition. The authors were able to link all 16 detected elements to various types of air pollutants and environmental exposures, as well as to reported results in the literature. An important observation was that areas which appeared dark in the visible light microscopy images did not correspond to regions of high-Z elemental concentrations. Moreover, the regions could not be easily identified in X-ray absorption images. This leads to the conclusion that microscopic regions affected by anthracosis are likely a low-density material, carbonaceous in nature, with the authors noting that “…the black-pigmented appearance is still elusive.”

The study of Carpentier et al. [93] focused on the chemical composition of Randall’s plaque—a mineral deposit at the surface of renal papilla and a nucleus for kidney stone formation. The major ingredients of Randall’s plaque are calcium phosphate apatite (CA) and amorphous carbonated calcium phosphate (ACCP). The authors compared Ca K-edge XANES spectra acquired from Randall’s plaque samples from two biopsies, biological CA, biological ACCP, and crystallized synthetic hydroxyapatatite (HAP). Comparison of the observed XANES features amongst these spectra led the authors to conclude that the spectra from ex vivo Randall’s plaque samples were mostly consistent with those of ACCP and that this chemical phase is a precursor of Randall’s plaque. Moreover, collecting the Ca K-edge XANES spectra at three points outside the visible part of Randall’s plaque, the authors also suggested that the ACCP-like form of the Ca depositions might occur within the renal papilla tissue, not only at its surface.

### 3.4. Hair, Skin, and Nails

The application of synchrotron radiation to the study of hair, skin, and nails has recently provided considerable insight surrounding questions of exposure, uptake, metabolism, and biokinetics of various elements. These types of tissue present the particular advantage of being relatively easy to access and, in the case of hair and nails, remove from living humans in significant quantities. Synchrotron microprobe capabilities were demonstrated and applied with human hair more than twenty years ago [94]. In this study, a 5 μm-sized beam was used to examine human hair in circular cross section. Hair was embedded within an epoxy resin, and when the resin hardened, sliced with a microtome at various locations along the growth axis in 10 μm sections. Elemental profiles for S, K, Ca, Fe, Cu, and Zn were obtained, and estimates of concentrations calculated. In the work of Martin et al. [95], a single hair from a lead smelter worker was examined using a similar synchrotron microprobe approach. In this example, the hair was cut with a blade and arranged in segments on kapton tape before synchrotron analysis. Clear signals were detected from K, Ni, Zn, and Pb, with lead (Pb) chosen for detailed study. The distribution of Pb suggested the external environment as the source of most of the Pb in this hair. A high profile synchrotron case involved the analysis of hairs belonging to Napoleon Bonaparte, with the goal of shedding light on the hypothesis that he was poisoned with arsenic [96]. For this study, multiple hairs were mounted in a frame and stepped through the beam using a three-stage translator. The results showed high levels of As, but with spatial profiles and concentrations which varied greatly from one hair to another, even when those individual hairs came from the same lock. This was inconsistent with the concept of an acute exposure from deliberate poisoning, and was suggestive of As contamination of the samples after death, perhaps through preservation efforts. This conclusion was reinforced by the observation of high concentrations of other toxic elements, including Hg, Pb, and Au. More recently, a study making use of X-ray fluorescence mapping, XAS/XANES speciation analysis, and other techniques, examined hair from an ancient human who had lived between 1000 and 1500 years ago in a region (in present-day Chile) known for high levels of geogenic As [97]. Inhabitants of the region continue to take in high levels of As, especially from drinking water, to the present day. Hair sections obtained from a mummy were measured both transversely and longitudinally. The distribution and chemical speciation of arsenic indicated a biogenic source (as opposed to external contamination introduced from bacteria or soil). Concentrations of As in the hair were extremely high (150 ppm). XANES suggested the presence of As(III) and As(V), mostly in the form of inorganic As species, but also as organic As compounds. The most prevalent species was found to be As(III) bound to S.

Synchrotron radiation applications with skin have produced a number of interesting and important results in recent years. An excellent example, which made use of both synchrotron X-ray fluorescence microscopy and EXAFS, was the work of George et al. [98]. This study examined the case of an individual with nephrogenic systemic fibrosis, a disease associated with exposure to Gd-based contrast agents. Autopsy skin tissues from the patient were analyzed in situ to learn about the distribution and chemical form of any Gd in the skin. Deposits containing Gd were determined, with the distribution of Gd found to correlate with Ca and P. EXAFS of the deposits revealed an absorption spectrum which differed from that of the original contrast agent, but was instead consistent with a GdPO_4_ structure. This represented the first direct evidence in human skin of a chemical release of Gd from contrast agents and subsequent uptake in a different chemical form. The capability of synchrotron radiation to explore the spatial characteristics of different elements was used in another recent study [99]. This investigation was also performed post mortem, but used punch biopsy skin cores taken from various locations (including thigh, back, chest, arm, and palm) on cadavers. The distribution of different elements as a function of depth was probed from the surface of the skin into the dermis. The beam size used was about 6 × 6 μm, with steps of 10 μm between measurements. A clear difference between concentrations in the epidermal and dermal layers were noted for Ca, Fe, and Zn, with higher elemental signals arising from the epidermis. Furthermore, the presence of Fe at the boundary of the epidermal and dermal layers was evident. Another recent paper used skin and lymph node tissue from human cadavers to investigate the biokinetics of tattoo pigments through their analysis with synchrotron X-ray fluorescence, XANES, and other methods [100]. In particular, Ti from the common white pigment of titanium oxide (TiO_2_) was targeted in this study, along with Br associated with a copper phthalocyanine green pigment. X-ray fluorescence maps were created with pixel sizes on the order of a micrometer, and XANES was performed over an energy range appropriate to investigate Ti speciation. Large tattoo particle groupings were determined from the μm-scale X-ray fluorescence mapping. XANES spectra indicated the presence of mostly rutile crystal structure TiO_2_, with minor contributions from anatase structure TiO_2_. A separate synchrotron run on a section of skin and lymph node tissue from a single cadaver used a much smaller beam size (50 nm) to determine TiO_2_ particle size. The mean tattoo particle size of TiO_2_ was estimated to be 180 nm. In another study, synchrotron radiation was used to probe the use of gold (Au) nanorods and the resulting distribution of Au relative to P and S in excised human skin [101]. Au nanorods are part of the ongoing development of nanoparticle dermal drug delivery systems, and their investigation is, therefore, important. Synchrotron samples were run in a high vacuum chamber to allow light elements to be studied, and the beam size used was very fine, allowing spatial area resolution of about 200 nm^2^. The spatial concentration of P and S in human skin were found to show an apparent “anti-correlation” with Au, suggesting an association between these elements and Au which shielded the lighter elements from detection. Future XANES applications were suggested to help clarify these elemental interactions.

Human nail clippings have gained increasing attention over the past decade with a series of synchrotron studies dedicated to their analysis. In one study, six clippings from six different donors were assessed using an EXAFS approach to determine the bonding geometry of Zn in nails [102]. Zn was found to be bonded with N and S, with four-fold coordination, and a ratio of S to N atoms in the first coordination shell of between 0.5 and 1.0 from the different clippings. Using a synchrotron beam size of 2 × 2 μm, the same group performed both XRF mapping and XANES to study Fe in nail clippings [103]. The distribution of Fe was determined to be clustered, with concentrations in the clusters up to six times higher than the other parts of the nail. Analysis of the XANES spectra indicated the presence of both Fe(III) and Fe(II) in octahedral bonding formations. Another study published at about the same time probed sections of two toenail clippings from children living in a historic gold mining area [104]. Since, in a geologic sense, arsenic (As) is associated with gold mineralization, this project sought evidence of As accumulation in toenail clippings using a microprobe beam of 10 × 10 μm. Indeed, As was detected and was localized mainly to the ventral and dorsal layers of the nail plate, with the highest concentrations in the ventral layer. Levels of As varied along the nail growth axis, suggesting differential levels of exposure or uptake over time. Analysis of XANES spectra revealed two species of As in the clippings: a lower oxidation state As(III) species, perhaps with mixed sulfur and methyl coordination; and a higher oxidation state As(V) species. More recently, Gherase et al. [30] examined As distribution in fingernail and toenail clippings from three healthy adult subjects. The beam size for these experiments was 28 μm × 10 μm, with a step size of 50 μm between point measurements. Maps of As distribution again revealed higher concentrations in the ventral and dorsal layers of the nail. Zn signal maps were also generated as part of the localization procedure for determining nail clipping boundaries. Some of these same nail clippings were used in a subsequent study to investigate As speciation [31]. XANES spectra from these clippings revealed As species to fall into three main groups: an As(III) type, fit as a mixture of As bound to thiols, and oxygen or methyl groups, with a small contribution from As(V) species; an As(V) type, best fit by arsenate in aqueous solution; and an As(III) plus As(V) mixture type. The indication that a high percentage of the As species were bound with sulfur was consistent with As binding with cysteine residues in keratin.

Another interesting application of synchrotron radiation of nails involved the assessment of a fingernail and a toenail from a deceased member of the Franklin Expedition [105], an ill-fated attempt to find the Northwest Passage sea route in the Canadian Arctic between 1845 and 1848. Longitudinal sections of the nails were cut with widths of 3 mm, and divided into pieces for various analyses. Synchrotron mapping was performed on the prepared and sectioned nail pieces using a beam size of 3–5 μm, with a 5 μm step size. Elements of interest included Cu, Zn, As, and Pb. The mapping provided unique insight into the time development of the uptake of various elements to the crew member’s nails. Results suggested that Pb exposure for the individual decreased over the sequence of the expedition, challenging the concept that widespread Pb intoxication may have played a role in the outcome of the voyage. Furthermore, it was indicated that Zn levels appeared to be especially low in the nails of this individual.

## 4. Conclusions

The rapid development of synchrotron-based XRF and XAS techniques and methods over the past few decades provided a new window into biology and medicine. Studies included in this review looked into molecular mechanisms behind many human diseases and conditions, existing or future treatment options, health effects of environmental or occupational exposures, and finding new biomarkers of adverse exposures.

Unprecedented sub-micrometer beam size and very intense beam delivery systems have allowed researchers to probe the distribution and chemical state of trace and major elements at the cellular and sub-cellular levels. Beyond the excellent spatial resolution, two main advantages of XRF and XANES are: (i) the ability to probe biological structures with minimally-invasive or -disruptive preparation, and, in some cases, no preparation and (ii) the penetrating nature of X-rays which overcomes the fundamental limitations of traditional visible light microscopy and scanning or transmission electron microscopy (SEM and TEM) methods.

A trend observed in the publications from the last several years is the coupling of XRF and XAS with other synchrotron-based methods such as Fourier transform infrared spectroscopy (FTIR), Raman spectroscopy, scanning transmission X-ray microscopy (STXM), X-ray magnetic circular dichroism (XMCD), X-ray diffraction (XRD), and ptychography or other phase-contrast imaging modalities. Each added measurement technique brings either different knowledge and/or information or further supports the conclusions drawn based on the other measurements. The compounded-method approach will likely continue in the future and brings about another important observation: many of the publications reviewed here represent the collective effort of large research groups or collaborations. Investigations of human or animal tissues and organs in their healthy or pathological states require multiple levels of expertise, training, and knowledge. If one also considers the required technical expertise of the synchrotron instrumentation, as well as the considerable effort and time needed, the trend of larger research teams appears reasonable.

A general conclusion of many studies included in this review was that abnormal distribution of trace elements was associated with pathology. However, in cases such as Parkinson’s and Alzheimer’s diseases, the precise origins of such abnormal accumulations or depletions of trace elements remain unknown. Whether the important scientific challenges highlighted in this paper will be met by synchrotron-based research efforts or not, one thing is certain: the studies reviewed here delivered significant contributions to the biological and medical sciences.

## Figures and Tables

**Table 1.** List of X-ray synchrotron-based methods used in trace elemental analysis of biological tissues.

Method	Acronym	Principles	References
X-ray Microtomography	μCT	Absorption contrast: differences in the x-ray linear attenuation coefficients. Phase contrast: interference of forward scattered x-rays. Contrast enhanced: addition of contrast agents to highlight targeted morphology.	Zehbe *et al.* [35] Betz *et al.* [36] Bournonville *et al.* [37]
Scanning Transmission X-ray Microscopy	STXM	X-ray microscopy technique in which a sample is scanned in the plane between an SR source and an x-ray detector. The plane is perpendicular to the x-ray beam direction and in the focal point of the x-ray lens.	Sakdinawat and Atwood [38]
X-ray Magnetic Circular Dichroism	XMCD	X-ray technique probing the magnetic state of materials. The measurement principle exploits the x-ray absorption differences between the left- and right-circularly polarized x-rays which arise from spin-orbit interactions of core-level atomic electrons.	van der Laan [39]
X-ray Ptychography (from Greek meaning ‘to fold’)	N/A	An iterative algorithm reconstructing amplitude and phase images from data sets obtained from the 2D detection of transmitted and forward-scattered x-rays from a scanning experiment.	Pfeiffer [40]
